# Discovering motifs that induce sequencing errors

**DOI:** 10.1186/1471-2105-14-S5-S1

**Published:** 2013-04-10

**Authors:** Manuel Allhoff, Alexander Schönhuth, Marcel Martin, Ivan G Costa, Sven Rahmann, Tobias Marschall

**Affiliations:** 1 Aachen Institute for Advanced Study in Computational Engineering Science (AICES), RWTH Aachen University, Germany; 2 Life Sciences Group, Centrum Wiskunde & Informatica, Amsterdam, Netherlands; 3 Bioinformatics, Computer Science XI, TU Dortmund, Germany; 4 Interdisciplinary Centre for Clinical Research (IZKF) & Institute for Biomedical Engineering, RWTH University Medical School, Aachen, Germany; 5 Genome Informatics, Faculty of Medicine, University of Duisburg-Essen, Germany

## Abstract

**Background:**

Elevated sequencing error rates are the most predominant obstacle in single-nucleotide polymorphism (SNP) detection, which is a major goal in the bulk of current studies using next-generation sequencing (NGS). Beyond routinely handled generic sources of errors, certain base calling errors relate to specific sequence patterns. Statistically principled ways to associate sequence patterns with base calling errors have not been previously described. Extant approaches either incur decisive losses in power, due to relating errors with individual genomic positions rather than motifs, or do not properly distinguish between motif-induced and sequence-unspecific sources of errors.

**Results:**

Here, for the first time, we describe a statistically rigorous framework for the discovery of motifs that induce sequencing errors. We apply our method to several datasets from Illumina GA IIx, HiSeq 2000, and MiSeq sequencers. We confirm previously known error-causing sequence contexts and report new more specific ones.

**Conclusions:**

Checking for error-inducing motifs should be included into SNP calling pipelines to avoid false positives. To facilitate filtering of sets of putative SNPs, we provide tracks of error-prone genomic positions (in BED format).

**Availability:**

http://discovering-cse.googlecode.com

## Introduction

Next-generation sequencing (NGS) technologies have tremendously influenced biomedical research. Thanks to its high speed and low cost, NGS has facilitated projects [1,2] that are based on tens of terabytes of sequencing data. Exome sequencing [3], which has been used in hundreds of studies [4], is even more cost-effective, as it limits itself to the medically most relevant coding regions. A major focus in most NGS-based studies are *single-nucleotide polymorphisms *(SNPs), many of which can be associated with phenotypic traits or diseases.

For all NGS platforms, cost efficiency and higher throughput come at the price of higher sequencing error rates. Beyond random miscalls, there are also systematic sources of errors. Since any base-calling error can be mistaken for a SNP, correcting for a maximum amount of (whatever kind of) sequencing errors is vital. In this study, we focus on a class of such errors that are characteristic for Illumina sequencing platforms [5]. On Illumina platforms, sequencing proceeds in cycles, where, in a rough sketch, during the *i*-th cycle, the *i*-th base of the fragment is read. Cycling can be confounded by various factors, which leads to *dephasing*: sequencing of partial amounts of fragments lags behind the overall sequencing procedure. Indeed, it is routine for Illumina sequencers to correct for mistakenly calling the (*i *+ 1)-th or (*i *- 1)-th base in the *i*-th cycle. Beyond this, higher error rates close to the end of reads [6] and an increase in miscalls in GC-rich regions [7] have been observed. Both of these error sources are likely to be related to dephasing. In the meantime, they have become common knowledge and are routinely handled.

In this work, we focus on a third kind of error, which, to date, has not yet undergone much principled investigation. In an initial study [8], it was pointed out that dephasing is also likely to be associated with specific sequence patterns. For example, large amounts of miscalls followed the nucleotide motif GGC and inverted repeats. It was hypothesized that dephasing may be due to backfolding of DNA (inverted repeats) and/or sequence-specific, preferential inhibition of RNA polymerase binding. Both phenomena are plausible and relate specific sequence patterns with delays in the cycling procedure. However, no rigorous framework for detecting those sequence patterns was provided in [8]. To overcome this limitation, Meacham et al. [9] developed a statistically principled framework, which combines hypothesis testing with logistic regression based classification. As an example, they report that the most common error-associated sequence context is GGT. The integration of (Phred-score based) read error profiles into the framework yields a gain in statistical power (recall) for detecting miscalls. However, it also leads to detection of sequence-unspecific error positions.

**Figure 1:**
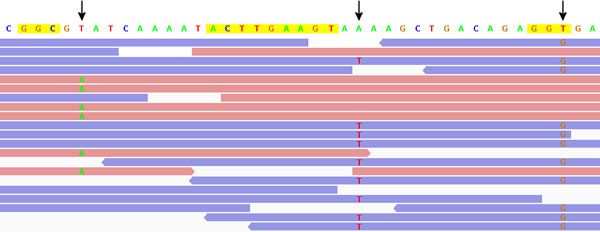
**Aligned reads with strand biased errors**. Hypothetical reads of two directions (red: forward; blue: backward) are aligned to a reference genome shown on top. Nucleotides within reads indicate mismatches to the forward reference. Three genome positions with extreme strand bias are marked by arrows. CSE-causing motifs described in [8] (GGC, inverted repeats) and [9] (GGT) are highlighted in yellow. Created with the Integrative Genomics Viewer (IGV) [18].

In the following, we refer to *systematic, truly sequence-specific errors *as *context-specific errors *(CSEs) and we refer to error-inducing sequence motifs as *contexts*. The method of choice to distinguish a CSE from a true SNP is to consider *strand bias*: Since a CSE is elicited by sequence patterns *preceding it, but not following it*, the error should be enriched in reads of one direction, but absent in reads of the other direction. Figure 1 illustrates such CSEs and their relationship with strand bias. We give a formal definition of strand bias in the Preliminaries section.

**Figure 2:**
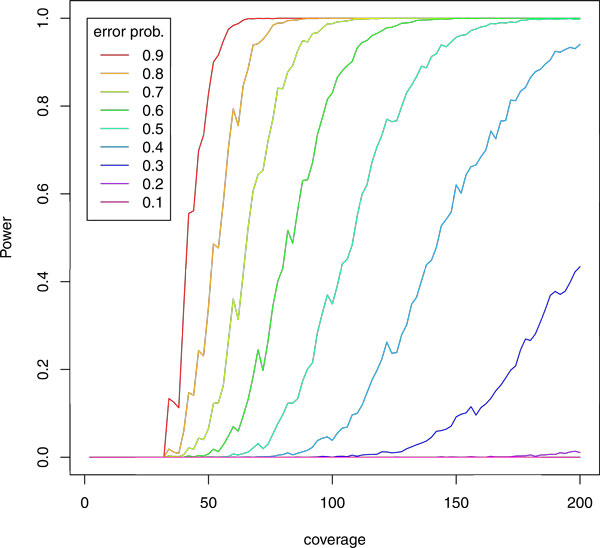
**Statistical power analysis**. Statistical power (probability, y-axis) to detect significant strand bias at a human exome position with Fisher's exact test, depending on read coverage (x-axis) and on assumed position-specific error rate (color) higher than the assumend background error rate of 0.01. Example: Even at an extremely high error rate of 0.5 (cyan), even a coverage of 100 grants only a discovery chance of 40%. Fluctuations are caused by finite sampling size for simulations.

Thanks to previous studies [8,9], detecting and filtering positions with strand bias has become routine. The Genome Analysis Toolkit (GATK) [10,11], for instance, annotates all putative SNPs with a p-value derived from a test for mismatch positions to be independent of read directionality (Fisher's exact test). This strategy, however, requires sufficient read coverage: If the coverage of a position is low, the statistical power for detecting whether that position is affected by strand bias is diminished, as illustrated in Figure 2.

Meacham et al. [9] also report substantial loss of power when considering statistics on *position-wise *read directionality, which motivates their above-mentioned integration of position-wise error profiles. This, however, while attaining good performance rates, also leads to potential confusion with sequence-unspecific errors, such as certain batch errors. In summary, none of the previous approaches provides a rigorous, statistically consistent framework to reveal sequence motifs as the very reason for base call errors. While [8] exclusively focus on CSEs, their approach for CSE detection is ad-hoc and, in fact, they report on suboptimal performance rates when distinguishing between true SNPs and CSEs. The GATK pipeline acts in a statistically principled manner, but has too little power where read coverage is low.

### Our approach

In this article, we propose a statistically principled procedure for CSE detection at maximum power. Our main idea is to pool genomic positions according to contexts. When screening pools for significant strand bias, we associate base calling errors with contexts, instead of single positions. This direct association is not only helpful in detecting relevant CSE-related sequences, but can also compensate for too low overall coverage, because pooling keeps statistical power at a high level. The detected motifs can serve for spotting error-prone positions already before the read mapping step. Last but not least, the motifs identified may also yield further insights into sequencing technology itself. Note also that our approach is generic and can be applied to any CSE-prone sequencing technology.

The article is organized as follows. In the Preliminaries section, we summarize a common SNP calling approach together with the statistical foundations to quantify strand bias. In the Algorithm section, we introduce our motif-based context discovery algorithm. We report the discovered sequence contexts in datasets from different Illumina sequencing platforms in the Results section, where we also investigate whether known SNPs (from dbSNP) are related to our discovered contexts. After that, we present a concluding discussion.

## Preliminaries

### Large-scale SNP analysis

SNPs are the most common kind of DNA mutation identified by the 1000 Genomes Project [1,12]. In diploid organisms, we distinguish between two types of SNPs. A homozygous SNP differs from a reference genome in both alleles, while a heterozygous SNP only differs in one allele. CSEs may be mistaken for heterozygous SNPs (if strand bias is not taken into account), as on average half of the reads differ from the reference.

We now briefly review a typical SNP calling pipeline. Read mappers usually align reads one by one, so they cannot make use of the information available through the set of all reads mapping to a locus. Therefore, tools such as the GATK [10,11] provide a local realignment algorithm for reads close to insertions and deletions, avoiding many wrong indel calls. Duplicates, i.e. reads which are derived from a single DNA fragment, are removed, since they do not give any additional evidence for or against a mutation and should not be considered independent in downstream analyses. The nucleotide (phred) quality scores given by the NGS device are recalibrated based on empirical probabilities. None of these steps is intended to detect or correct context-specific errors. We also remark that more accurate base-calling algorithms do not prevent CSEs [9].

Each (sufficiently covered) genome position is tested for a SNP as follows. A *pileup *is produced (i.e., a list of all reads covering the position), from which a 2 × 2 contingency table is derived, containing the number of matches/mismatches of forward/backward reads at that position (see Table 1). The GATK avoids wrong SNP calls due to CSEs by testing each putative SNP position for strand bias using Fisher's exact test (see the following section). The plausibility of a true SNP decreases with increasing strand bias. The coverage at a position determines the statistical power to detect significant strand bias and, thus, the above procedure can fail to detect strand bias in regions of low coverage.

**Table 1:** 2 × 2 contingency table

	Match	Mismatch	Total
Forward	*a*	*b*	*f*
Backward	*c*	*d*	*k*

Total	*m*	*s*	*n*

2 *× *2 contingency table; *a, b, c, d*: numbers of reads; *f, k, m, s*: marginals; *n *= *a *+ *b *+ *c *+ *d *= *f *+ *k *= *m *+ *s*.

### Fisher's exact test

Fisher's exact test computes a p-value from a 2 × 2 contingency table (Table 1) to decide whether the two data characteristics "read direction" and "mismatch fraction" are independent, which is equivalent to testing whether the rows have the same distribution. If this is the case, then there is no evidence for strand bias.

To calculate the p-value of Table 1, Fisher's test assumes that all marginals are fixed and given. We write M=(f,k,m,s,n), where *n *= *f *+ *k *= *m *+ *s *to denote the marginal information. Given  M and one entry (without loss of generality, we choose *a*), we can compute all further table's entries, and we write (a|M) for such a representation of Table 1. The null hypothesis probability ${\text{Pr}}_{H_{0}}\left(a|M\right)$ of an observed table (a|M) is

$$\text{P}{\text{r}}_{H_{0}}\left(a|M\right)=\frac{\left(\begin{array}{c} a+b\\ a \end{array}\right)\;\cdot \;\left(\begin{array}{c} c+d\\ c \end{array}\right)}{\left(\begin{array}{c} a+b+c+d\\ a+c \end{array}\right)}.$$

The p-value of (a|M) is the probability of observing this or a more extreme table under the null hypothesis. A more extreme table means a table with a lower probability than the observed one.

$$\text{p-}\text{value}(a\text{|}M)\;=\;\sum_{a\prime \in E(a,M)}Pr_{H_{0}}(a\prime \text{|}M),$$

where the "extreme" values of *a' *are from the set $E\left(a,M\right):=\;\left\{a^{\prime }:{\text{Pr}}_{H_{0}}\left(a^{\prime }|M\right)\leq {\text{Pr}}_{H_{0}}\left(a|M\right)\right\}$. If the p-value is sufficiently low, we reject the null hypothesis, meaning we assume that the two rows were not sampled from the same distribution. The quantity - log_10_(p-value(a|M)) can be considered as a quantitative measure of strand bias and is called the *strand bias score*.

Fisher's exact test is computationally expensive for tables with large entries, but can be replaced by a χ^2 ^test in this case [13].

### Multiple testing

When many statistical tests are performed, the expected number of false positives can also be large. There are many strategies to deal with such situations of *multiple hypotheses testing*. One popular approach, for instance, is to control the *false discovery rate *(FDR) as advocated by Benjamini and Hochberg [14]. Another option is to control the *family-wise error rate *(FWER) by means of a Bonferroni correction, that is, controlling the probability of making at least one type I error among all tested hypotheses, which is more conservative than controlling the FDR (at the same level). In our case, p-values of significant motifs are very low due to the large amounts of available NGS data and, in particular, due to our pooling strategy. Therefore, we can opt for the more conservative Bonferroni correction without losing many significant motifs.

### Power considerations

We analyse the power of Fisher's exact test by a sampling procedure, resulting in Figure 2.

To this end, we pick an error probability *e *(color-coded in Figure 2, between 0.1 and 0.9) and a coverage *n *(x-axis in Figure 2, between 2 and 200 with a step size of 2) for a simulation. We assume that there is a constant low background error probability unrelated to CSEs, here set to *ε *= 0.01.

We draw *n*/2 samples to obtain the "forward" row of Table 1 using a mismatch probability of *e*; that is, on average, we obtain *a *= (1 - *e*)*n*/2 and *b *= *en*/2. We draw further *n*/2 samples (for a total coverage of *n*) to obtain the "backward" row of the table using a mismatch probability of *ε*; i.e., on average, *c *= (1 - *ε*)*n*/2 and *d *= *εn*/2. This restricts the sampled tables to those with equal row sums, which is sufficient for our illustration.

We perform Fisher's exact test and record whether we reject the null hypothesis (as we should, since *e *≫ *ε*) at *α *= 0.05/(5 · 10^7^), a Bonferroni-corrected test level for the human exome. We repeat the sampling experiment *T *= 3000 times. The empirical power is the fraction of times that we reject the null hypothesis out of these *T *repetitions.

Figure 2 clearly shows that at low coverages, it is almost impossible to detect significant strand bias. The power curves are improved by choosing less conservative thresholds, but in the end, only high coverage guarantees statistical power.

## Algorithm

### Motif space

Given a set of reads aligned to a reference genome, our aim is to discover *motifs *that cause sequencing errors. We model motifs as *generalized strings*, that is, sequences of sets of characters allowed at each position. Such character sets are usually abbreviated by IUPAC characters, e.g. N stands for aNy character and thus corresponds to the set {A, C, G, T}. The motif GNT, for example, matches the strings GAT, GCT, GGT, and GTT. To limit the number of hypotheses to test, we only allow the wildcard N (and no other IUPAC wildcards). We assume that the motif length *q *and the maximal number *n *of allowed Ns are given as input parameters. Let S(q,n) be the resulting motif space. Thus, the size of S(q,n) is

$$S\left(q,n\right):=|S\left(q,n\right)|\;=\;\overset{n}{\underset{i=0}{\sum }}\left(\begin{array}{c} q\\ i \end{array}\right)\cdot 4^{q-i},$$

the *i*-th term of the sum giving the number of motifs with *i *Ns. Conceptually, we want to perform one strand bias test as described in the Preliminaries section for each motif in the motif space S(q,n), omitting motifs that do not occur in the given reference genome.

### Contingency table construction

For a given motif *m*, we locate all occurrences of *m *in the reference genome and its reverse complement. From pileups, we costruct an aggregated contingency table, whose entries we name *a, b, c, d *as in Table 1. This is in principle straightforward, but some care is required to keep track of the two genome strands and both read directions. The pileup position (i.e., the position of interest) is defined to be the last position of the motif. This is not a severe restriction when a sufficient number of wildcards is allowed.

**Figure 3:**
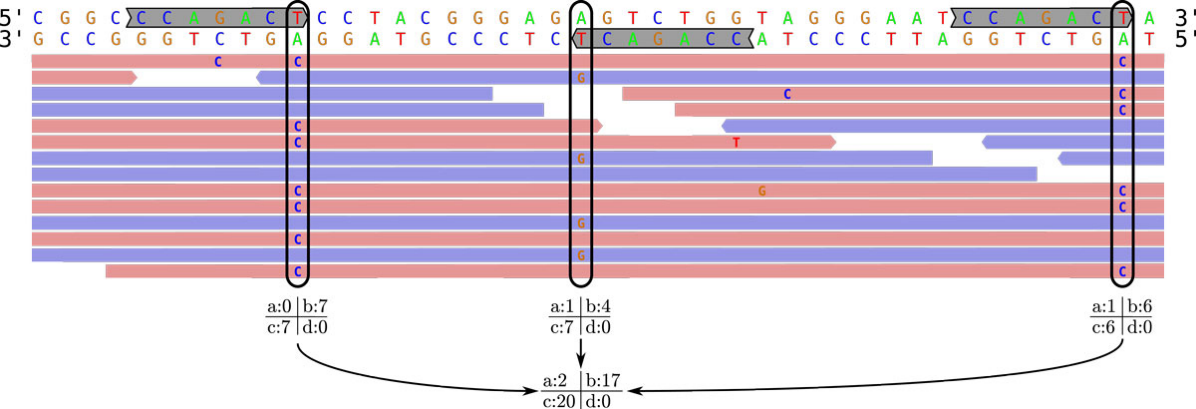
**Contingency table construction for the motif **CCAGACT. Contingency table construction for the motif CCAGACT. The forward reference (5*' *to 3*'*) is displayed at the top; below, its complement is shown (3*' *to 5*'*). F-reads are indicated as red arrows, R-reads as blue arrows. F-intervals are marked in the forward reference, R-intervals are marked in the reverse complementary reference. Two F-positions (last position in an F-interval) and one R-position (first position in an R-interval) are indicated by vertical boxes. The corresponding individual contingency tables and the resulting joint contingency for the motif CCAGACT are shown below the alignments. Note that F-positions and R-positions both contribute to the motif's contingency table, as described in the Algorithm section.

Let us call a genomic interval where the motif *m *matches the forward (resp. reverse complementary) reference an *F-interval *(resp. *R-interval*), and the last (resp. first) position of an F-interval (resp. R-interval) an *F-position *(resp. *R-position*). Genomic positions *i *always refer to the forward reference. Further, let us call a read that has been mapped to the forward (resp. reverse complementary) reference an *F-read *(resp. *R-read*). Figure 3 provides an illustration.

A *match *occurs at position *i *if either the read is an F-read and the nucleotide of the forward reference at position *i *equals the aligned read nucleotide, or if the read is an R-read and the forward reference at position *i *equals the *complement *of the aligned read nucleotide. In all other cases, a *mismatch *occurs. As a convention, in a pileup, the nucleotides of R-reads have been already complemented, so a pileup column can always be compared to the forward reference. At the single R-position in Figure 3, while the pileup indicates an A → G mismatch in many of the R-reads, this is in fact technically a T → C mismatch because of this convention. All three positions shown in this hypothetical example would therefore consistently indicate strand bias and hence a CSE (more precisely, a consistent T → C miscall after CCAGAC).

We compute the motif's contingency table entries as follows:

• Initialize *a *= *b *= *c *= *d *= 0.

• For every F-position of motif *m*, we obtain a pileup, and we increment *a *by the number of matching F-reads, *b *by the number of mismatching F-reads, *c *by the number of matching R-reads, and *d *by the number of mismatching R-reads in each pileup.

• For every R-position of motif *m*, we obtain a pileup, and we increment *a *by the number of matching R-reads, *b *by the number of mismatching R-reads, *c *by the number of matching F-reads, and *d *by the number of mismatching F-reads in each pileup.

Note that in some cases, a genome position may be both an F-position and an R-position, such as for the motif GGN and the reference ...GGACC..., where this occurs at the A.

### Context discovery algorithm

We now describe our algorithm to discover contexts inducing CSEs. The input to the algorithm consists of a reference genome, a collection of aligned reads, a motif length *q*, a maximal wildcard number *n*, an FWER threshold *α >*0, a background error rate threshold *ε >*0 and an error rate difference (ERD) cutoff *δ >*0. Typical values are *q *≤ 10, *n *≤ *q/*2, *α *= 0.05, *ε *= 0.03, *δ *= 0.05.

We first compute the Bonferroni threshold T:=α/|S(q,n)|. By a single linear pass through the reference genome (computing its reverse complement locally on the fly), we incrementally compute the contingency tables of each *q*-gram (a DNA sequence of length *q *without wildcards). All contingency tables are stored in a hash map; the *q*-gram sequence is mapped to its contingency table. For each motif m∈S(q,n), we now obtain the contingency table of *m *by adding the tables of all *q*-grams that match *m*. We apply Fisher's test and compute the strand bias p-value and score and deem all motifs with a p-value lower than or equal to the Bonferroni threshold *T *significant.

For each significant motif, we compute the forward error rate FER = *b/*(*a *+ *b*) and the reverse error rate RER = *d/*(*c *+ *d*) in terms of the contingency table (Table 1). We remove motifs with RER *≥ ε *(i.e., above the typical background error rate threshold). For the remaining motifs, we compute the error rate difference ERD = FER *- *RER. We remove those motifs with too small ERD to be of interest, i.e., those with ERD *< δ*.

We sort the remaining motifs by decreasing ERD and report them with their properties. We opted for sorting the motifs by ERD (instead of by strand bias score), because a higher score does not imply that the motif is more likely to cause sequencing errors. A higher score (i.e., lower p-value) may simply reflect that the motif occurs more often in the genome or is covered by more reads, which grants higher statistical power and lower p-values.

Note that some of the best motifs (with highest ERD) may be similar to each other and do not provide fundamentally new information. We choose to report all motifs initially, as the resulting list may be postprocessed by comparing motifs depending on the situation.

## Computational results

### Datasets and parameters

We analyse four datsets from three different Illumina platforms: GAIIx, MiSeq and HiSeq2000. Table 2 gives details, and from now on we refer to the datasets by the names given in that table. Datasets were chosen according to the following considerations. To be able to relate our results to those of Nakamura et al. [8], we include their dataset (GAIIx-bs), which consists of reads from *Bacillus subtilis *sequenced on the GAIIx platform. We additionally analyse reads from the human genome due to its prevalence in many studies. First, we include a GAIIx dataset (GAIIx-hg) from the 1000 Genomes project [1], from which we consider chromosome 1. For the newer HiSeq platform, we include another 1000 Genomes project dataset (HiSeq-hg). MiSeq is a benchtop platform allowing for fast and easy sequencing of smaller datasets, making it interesting also for validation of SNP calls. Therefore, the question whether it is prone to the same CSEs as GAIIx and HiSeq is of great interest. As we presently do not have any MiSeq datasets on the human genome, we include *Escherichia coli *reads provided by Illumina (MiSeq-ec).

**Table 2:** Overview of datsets

Name	Organism	Reads Accession	Genome Accession	**Ref**.
GAIIx-bs	*B. subtilis str. 168*	DRA DRX000504	NCBI NC_000964.3	[8]
GAIIx-hg	Human chr. 1	HG00131	GRCh37	[1]
MiSeq-ec	*E. coli *DH10B	Illumina (*)	NCBI NC_010473.1	
HiSeq-hg	Human chr. 1	HG00108	GRCh37	[1]

Overview of Datsets. Names refer to Illumina platform and organism. DRA: DDBJ sequence read archive.

Illumina reads (*): http://www.illumina.com/systems/miseq/scientific_data.ilmn.

We expect the discovered motifs to be platform-dependent rather than genome-dependent and, thus, to also cause CSEs when sequencing another genome. The power to detect CSE-causing motifs depends on the number of their occurrences in the used genomes and we might thus miss motifs for MiSeq that are not frequent in *E. coli*.

We use BWA version 0.5.9-r18-dev with standard parameters for read mapping [15]. As described in the Preliminaries section, SNP discovery pipelines spend considerable computational effort on cleaning up the initial mapping by re-aligning reads around gaps, removing duplicates, and recalibrating quality values. We examine how strongly these steps influence the discovered contexts. As we report in section "Effects of postprocessing", we find that they do not strongly influence the results from a qualitative point of view. Therefore, the results reported in the following section are based on read mapping only, without additional postprocessing steps.

Our context discovery algorithm is run with different parameter settings on the motif length *q *and number of wildcards *n*; to assess a broad range of possible motifs we analyse all datasets with the two combinations (*q, n*) = (8, 4) and (*q, n*) = (4, 1). The setting (4, 1) was chosen to compare our findings with previously reported motifs which are frequently of type (3, 0), while the setting (8, 4) aims at examining whether there exist more specific motifs that cause CSEs. These two resulting motif spaces are quite different in size, |S(4,1)|=512 and |S(8,4)|=386560, and thus resemble different trade-offs between flexibility and excessive multiple testing. We also applied our algorithm using (*q, n*) = (10, 2). While the corresponding results did not undergo a thorough analysis, we display some of the highlights below and provide the full lists as additional files 6, 7, 8, 9.

### Discovered sequence contexts

We ran the algorithm described above for each dataset and parameter setting detailed in the previous section (8 combinations in total). To control for multiple testing, we used a FWER cutoff of *α *= 0.05. Only motifs with a "normal" RER were retained during filtering, employing a RER cutoff of *ε *= 0.03. For motif space (8,4), we set *δ *= 0.1 to only retain motifs with an ERD of at least 10 percent. For the shorter motifs (4,1), this setting yielded no motifs after filtering; so we applied a less stringent filtering at *δ *= 0.01. This indicates that with longer, i.e. more specific, motifs we are indeed able to discover contexts causing higher error rate differences than shorter motifs that have exclusively been considered in the literature until now. All filter cutoffs and the numbers of motifs after filtering are summarized in Table 3.

**Table 3:** Overview of filter settings

Search space	Thresholds			Number of motifs per dataset			
	**FWER**	**RER**	**ERD**	**GAIIx-bs**	**GAIIx-hg**	**MiSeq-ec**	**HiSeq-hg**

(4,1)	*α *= 0.05	*ε *= 0.03	*δ *= 0.01	4	0	6	5
(8,4)	*α *= 0.05	*ε *= 0.03	*δ *= 0.1	8	13	26	74

Overview of filter settings and number of remaining significant motifs after filtering per dataset and per search space.

The discovered contexts are given in Table 4, together with the respective contingency table entries, strand bias score, forward/reverse error rate, and error rate difference. For (4,1), the table contains all motifs meeting our filter criteria. For the sake of clarity, it only includes a selection of motifs from the (8,4) category, while the full set is provided as additional files 1, 2, 3, 4. Not suprisingly, many motifs are similar to each other as we did not take any measure to avoid redundancy. Instead, we decided to give the full set as additional files and try to give a representative selection in Table 4. In the following, we discuss our findings separately for (4, 1) and (8, 4).

**Table 4:** A selection of CSE-causing motifs

(*q, n*)	Context	**Rk**.	**Occ**.	FM = *a*	RM = *c*	FMM = *b*	RMM = *d*	- log(p)	FER [%]	RER [%]	ERD [%]
**Dataset: GAIIx-bs**											
(8, 4)	NGGCGGGT	3	264	5857	6867	859	40	180.0	12.8	0.6	12.2
	CGGNGGGT	4	136	3366	3930	477	22	121.2	12.4	0.6	11.9
	GGCGGGGT	5	62	1318	1624	180	5	52.0	12.0	0.3	11.7
	ACGGCGGG	6	84	1690	2065	241	17	58.3	12.5	0.8	11.7
(4, 1)	GGGT	1	13478	374933	384732	10002	2643	∞	2.6	0.7	1.9
	CGGT	2	25144	716801	730328	14765	5071	∞	2.0	0.7	1.3
	AGGT	3	20146	581562	584578	12086	4237	∞	2.0	0.7	1.3
	NGGT	4	79810	2272988	2317196	46304	16224	∞	2.0	0.7	1.3

**Dataset: GAIIx-hg**											
(8, 4)	CGGCGGGT	1	532	731	1330	169	7	60.7	18.8	0.5	18.3
	TGGCGGGT	2	3232	5715	6410	1128	37	229.3	16.5	0.6	15.9
	CGGCAGGT	3	1396	2788	3522	409	19	110.8	12.8	0.5	12.3
	NGGCGGGT	10	13712	24040	30886	3029	158	∞	11.2	0.5	10.7
(4, 1)	No motifs passed filter										

**Dataset: HiSeq-hg**											
(8, 4)	TGGCGGGT	1	3232	3803	5547	1475	53	∞	27.9	0.9	27.0
	CGGCGGGT	2	532	418	777	152	4	56.1	26.7	0.5	26.2
	CGGCAGGT	4	1396	1935	2820	567	23	167.5	22.7	0.8	21.9
	NGGCGGGT	10	13712	17251	26924	4432	177	∞	20.4	0.7	19.8
	GTGGCTTG	17	7568	12047	18583	2526	67	∞	17.3	0.4	17.0
(4, 1)	GGGT	1	1366400	3208669	3340323	82048	15104	∞	2.5	0.5	2.0
	AGGT	2	1836218	4530889	4740634	87166	20448	∞	1.9	0.4	1.5
	NGGT	3	5261516	13265123	13614878	239748	57694	∞	1.8	0.4	1.4
	CGGG	4	460830	876560	861233	16336	4710	∞	1.8	0.5	1.3
	CGGT	5	232662	516547	521942	9306	2544	∞	1.8	0.5	1.3

**Dataset: MiSeq-ec**											
(8, 4)	GGCGGGGT	1	102	16780	24956	5809	88	∞	25.7	0.4	25.4
	GGCGCCTC	4	4	349	506	84	1	28.7	19.4	0.2	19.2
	NGGCGGGT	5	762	122922	171199	28401	879	∞	18.8	0.5	18.3
	CGGNGGGT	11	444	74979	95226	12415	568	∞	14.2	0.6	13.6
	CGGCGGGN	12	942	158741	205881	25090	1187	∞	13.6	0.6	13.1
(4, 1)	GGGT	1	24802	5324301	5495475	145090	24701	∞	2.7	0.4	2.2
	AGGT	2	27414	5979767	6104684	121330	29230	∞	2.0	0.5	1.5
	NGGT	3	146116	32813986	33422161	604790	162298	∞	1.8	0.5	1.3
	CGGT	4	49530	10934765	11081037	184200	54762	∞	1.7	0.5	1.2
	GGGN	5	78504	20903313	21323544	338589	114360	∞	1.6	0.5	1.1
	CGGG	6	32740	7089342	7227334	115433	42523	∞	1.6	0.6	1.0

A selection of CSE-causing motifs for each combination of dataset and parameters. For each motif, we give the rank (Rk.) in the original list sorted by ERD; number of occurrences in the respective genome (Occ.); the contingency table entries FM, RM, FMM, and RMM; the forward error rate FER = FMM/(FM + FMM); the reverse error rate RER = RMM/(RM + RMM); and the error rate difference ERD = FER *- *RER. If a motif's p-value cannot be numerically distinguished from zero within double precision, we report a - log(*p*) score of ∞.

#### Motif space (4,1)

We included motif space (4,1) to compare our results to previous findings. As discussed in the introduction, the 3-grams GGC and GGT have been reported to be linked to CSEs. Table 4 shows that we indeed find strong evidence for a significantly biased error distribution at GGT sites for datasets GAIIx-bs, HiSeq-hg, and MiSeq-ec. However, the observed difference in error rates at such sites is quite low, ranging from 1.2 to 2.0 percent, and therefore these motifs alone will most likely not disrupt SNP calling. The motif GGC does not appear in the (4,1)-results. By looking at the (8,4)-results, we see that GGC is associated to CSEs, but usually appears 4 base pairs before the first error site. Our analysis also reveals the motif CGGG that appears in the result list for HiSeq-hg and MiSeq-ec.

#### Motif space (8,4)

The observed ERD values for (8,4)-motifs are consistently larger than for (4,1)-motifs by approximately one order of magnitude. This shows that our algorithm is able to discover longer, more specific and informative motifs. For many motifs, especially for the HiSeq and MiSeq platforms, ERD is around 20 percent. Motif NGGCGGGT, for instance, leads to forward errors rates of 20.7 percent on HiSeq instruments, while the reverse error rate is 0.7, which is a normal value for this platform. This particular motif was discovered in all four datasets. In general, the found motifs were quite consistent across platforms and datasets, showing that, on the one hand, Illumina sequencers share common characteristics and, on the other hand, that our algorithm robustly detects CSE-causing motifs. Such specific high-ERD CSE-causing motifs have never been reported in the literature before.

#### Remark on Motif Space (10,2)

While we do not analyse motifs from (10,2) in detail here, it is notable that some of such motifs discovered in the HiSeq-hg dataset have a FER larger than 0.5, implying that base calls at corresponding positions are more likely to be erroneous than to be correct. These motifs may serve as points of entrance for examining machine protocols. See additional files 6, 7, 8, 9 for full lists of all (10,2)-motifs for all four datasets.

### Effects of postprocessing

To assess the effects of postprocessing steps after read mapping, as usually done in SNP calling pipelines such as that of the GATK, on the contexts we discover, we here report results on dataset GAIIx-bs. After read mapping, we apply the local realignment step of GATK version v2.2-15-g4828906 and delete duplicates with samtools [16]. For (4, 1), exactly the same motifs as reported in Table 4 were discovered when using this additional alignment postprocessing. For (8, 4), we discovered 74 motifs with postprocessing (the top 10 of which are shown in Table 5) instead of only 8 motifs without it (see additional files 1 and 5). The duplicate removal step appears to have a significant effect on the FER and hence on the ERD, such that many more motifs reach the treshold of *δ *= 10%. The 74 motifs appear closely related to each other and to those 8 discovered without postprocessing, so the results are, from a qualitative standpoint, comparable. To summarize, postprocessing does neither prevent CSEs (and does not intend to) nor does it fundamentally change the discovered contexts.

**Table 5:** Top 10 discovered motifs after alignment postprocessing

Rank	Context	FER [%]	RER [%]	ERD [%]
1	ACGGCGGT	26.1	0.5	25.6
2	GTGGCGGT	25.1	0.7	24.4
3	GCGGCGGT	22.9	0.7	22.2
4	GTGGCTGT	22.4	0.6	21.8
5	ATGGCGGT	21.2	1.0	20.3
6	NCGGCGGT	20.0	0.7	19.3
7	GTGGCTTG	20.2	1.2	19.0
8	GNGGCGGT	19.2	0.7	18.5
9	GCGGCTGT	18.8	0.7	18.1
10	ACGGCTGT	18.6	0.8	17.7

Top 10 (based on ERD) contexts on dataset GAIIx-bs with (*q, n*) = (8, 4) after GATK postprocessing and duplicate removal.

### Effects on SNP calling

Today, detecting SNPs is routinely done and millions of SNPs have been collected in databases like db-SNP [17]. In the following, we discuss to what extend SNP calling is influenced by error-causing motifs. To identify known SNPs that might be difficult to call using Illumina technology, we pool all (8,4)-motifs discovered in the four datasets. The resulting 91 motifs yield 6 622 827 putatively error-prone positions on the human genome (0.21% of all positions). Of these, 82 684 are co-located with SNPs in dbSNP build 137; that means, 0.29% of all 28 440 783 SNPs in the database lie at error-prone positions. Although the fraction is small, an absolute number of 82 684 SNPs that are difficult to genotype using Illumina devices is still remarkable. The difference between 0.21% of all genomic positions and 0.29% of all SNPs is small, but it is statistically significant (*p <*10*^-^*^15 ^according to a *χ*^2^-test). However, whether this difference is due to false positives caused by CSEs or due to other effects remains open.

As a next step, we called SNPs in the two human datasets GAIIx-hg and HiSeq-hg to test whether they are enriched for CSE-prone positions. For SNP calling, we used the GATK's UnifiedGenotyper with default parameters. Datasets GAIIx-hg and HiSeq-hg yielded 2 525 553 and 2 609 149 SNPs, respectively; out of these, 9 126 (0.36%) and 14 844 (0.57%) were found at CSE-prone positions. Thus, the fractions of SNPs at such positions are clearly higher than the corresponding fraction in dbSNP, which might indicate that the set of called SNPs does indeed contain false positives that are due to CSEs.

## Discussion and conclusion

We have presented an algorithm to identify sequence contexts that cause context-specific sequencing errors (CSEs). In contrast to previous approaches, which detect *positions *with strand bias and then report on common sequence motifs at the identified positions, we start from the *motifs *and aggregate information at matching positions, which grants much higher statistical power. Our approach is thus able to integrate many weak but consistent positional signals. Allowing wildcards within the sequence motifs grants additional flexibility, e.g. the motifs with (*n, q*) = (4, 1) will also discover contexts of length 3.

Our approach is the first motif-based CSE discovery method. We confirm previously reported error-prone sequence contexts [8,9] but also find much more informative motifs with an ERD higher by one order of magnitude. This allows to exactly pinpoint problematic positions, while the previously known short contexts GGT and GGC do not reliably predict strongly CSE-prone positions. The approach is also robust in the sense that extracted motifs were closely related across datasets.

The practical significance of error context discovery lies in the fact that an increasing number of exome sequencing studies to identify genetic causes of Medelian diseases and genome-wide association studies depend on reliable SNP calling. Our work can be integrated as an additional step into SNP calling pipelines, down-weighting proposed SNPs at known error contexts for the platform, independently of the coverage and strand bias in the particular dataset under investigation. To facilitate filtering of SNPs, we provide platform-specific annotation tracks (in BED format) with positions in the human genome matching discovered contexts. Our implementation is available under the terms of the GNU General Public License. Annotation tracks and source code can be obtained from the URL given in the Abstract.

We plan to systematically compare discovered contexts on more datasets from different organisms, sequenced on the same platform and with more parameter combinations (*q, n*) and with additional IUPAC wildcards (beyond N) to quantify the robustness of motif-based approaches comprehensively. We will also extend our algorithm to contexts on both sides of error-prone positions, with a special emphasis on inverted repeats. Furthermore, we plan to provide error contexts and annotation tracks for other (non-Illumina) sequencing platforms.

## Competing interests

The authors declare that they have no competing interests.

## Supplementary Material

Additional File 6 **Full list of (10,2)-motifs for dataset GAIIx-bs**. All (10,2)-motifs found in dataset GAIIx-bs (after filtering). Click here for file

Additional File 7 **Full list of (10,2)-motifs for dataset GAIIx-hg**. All (10,2)-motifs found in dataset GAIIx-hg (after filtering). Click here for file

Additional File 8 **Full list of (10,2)-motifs for dataset MiSeq-ec**. All (10,2)-motifs found in dataset MiSeq-ec (after filtering). Click here for file

Additional File 9 **Full list of (10,2)-motifs for dataset HiSeq-hg**. All (10,2)-motifs found in dataset HiSeq-hg (after filtering). Click here for file

Additional File 1 **Full list of (8,4)-motifs for dataset GAIIx-bs**. All (8,4)-motifs found in dataset GAIIx-bs (after filtering). Click here for file

Additional File 2 **Full list of (8,4)-motifs for dataset GAIIx-hg**. All (8,4)-motifs found in dataset GAIIx-hg (after filtering). Click here for file

Additional File 3 **Full list of (8,4)-motifs for dataset MiSeq-ec**. All (8,4)-motifs found in dataset MiSeq-ec (after filtering). Click here for file

Additional File 4 **Full list of (8,4)-motifs for dataset HiSeq-hg**. All (8,4)-motifs found in dataset HiSeq-hg (after filtering). Click here for file

Additional File 5 **Full list of (8,4)-motifs for dataset GAIIx-bs after alignment postprocessing**. All (8,4)-motifs found in dataset GAIIx-bs with alignment postprocessing. Click here for file

## Declarations

Publication of this article was funded by RWTH Aachen and CWI Amsterdam.

This article has been published as part of *BMC Bioinformatics *Volume 14 Supplement 5, 2013: Proceedings of the Third Annual RECOMB Satellite Workshop on Massively Parallel Sequencing (RECOMB-seq 2013). The full contents of the supplement are available online at http://www.biomedcentral.com/bmcbioinformatics/supplements/14/S5.
